# Remote Sensing-Derived Environmental Variables to Estimate Transmission Risk and Predict Malaria Cases in Argentina: A Pre-Certification Study (1986–2005)

**DOI:** 10.3390/pathogens14050448

**Published:** 2025-05-01

**Authors:** Ana C. Cuéllar, Roberto D. Coello-Peralta, Davis Calle-Atariguana, Martha Palacios-Macias, Paul L. Duque, Liliana M. Galindo, Mario O. Zaidenberg, María J. Dantur-Juri

**Affiliations:** 1 National Veterinary Institute, Technical University of Denmark, Bülowsvej, 2750 Frederiksberg, Denmark; anacarocuellar@gmail.com; 2 Department of Microbiology, Faculty of Veterinary Medicine and Zootechnics, Universidad de Guayaquil, Guayaquil 090511, Ecuador; roberto.coellope@ug.edu.ec (R.D.C.-P.); davis.callea@ug.edu.ec (D.C.-A.); martha.palaciosm@ug.edu.ec (M.P.-M.); 3 Unidad Ejecutora Lillo (CONICET-Fundación Miguel Lillo), San Miguel de Tucumán 4000, Argentina; polduquebiologo@gmail.com; 4 Facultad de Medicina, Universidad Nacional de Tucumán, San Miguel de Tucumán 4000, Argentina; galindolilianamaria@gmail.com; 5 Coordinación Nacional de Control de Vectores, Ministerio de Salud de la Nación, Salta 4400, Argentina; mozainderberg@gmail.com; 6 Instituto de Genética y Microbiología, Fundación Miguel Lillo, San Miguel de Tucumán 4000, Argentina

**Keywords:** malaria, predictive models, satellite images, ARIMA temporal series, disease prevention

## Abstract

Early warning systems rely on statistical prediction models, with environmental risks and remote sensing data serving as essential sources of information for their development. The present work is focused on the use of remote sensing for the estimation of transmission risk and the prediction of malaria cases in northwest Argentina. This study was conducted in the city of San Ramón de la Nueva Orán, where cases of the disease have been reported from 1986 to 2005. The relationship between reported malaria cases and climatic/environmental variables—including the normalized difference vegetation index (NDVI), normalized difference water index (NDWI), and land surface temperature (LST)—obtained from Landsat 5 and 7 satellite images was analyzed using multilevel Poisson regression analyses. An increased abundance of reported malaria cases was observed in summer. An ARIMA (autoregressive integrated moving average) temporal series model incorporating environmental variables was developed to forecast malaria cases in the year 2000. The analysis of the relationship between malaria cases and environmental and climatic factors showed that malaria cases were associated with increases in LST and mean temperature and a decrease in the NDVI. Early warning systems that provide information about spatial and temporal predictions of epidemics could help to control and prevent malaria outbreaks. Based on these findings, this study is expected to support the development of future prevention and control measures by health officials.

## 1. Introduction

The application of remote sensing and Geographic Information Systems (GIS) to epidemiological events provides an integrated approach to disease monitoring and introduces new possibilities for their control. These technologies enable the monitoring of environmental factors that are crucial for the transmission of malaria, such as temperature, rainfall, and vegetation, thus improving surveillance and targeted interventions [1,2]. Early warning systems that provide information about temporal and spatial predictions of epidemics, including the increased frequency and intensity of outbreaks driven by climate-related environmental changes, can support prevention efforts [3,4]. As these systems ensure that health authorities and decision-makers are aware of immediate threats and are prepared to take effective measures, they enable the strategic prioritization of resources in vulnerable areas that are exposed to heightened climate-related health risks [5,6,7]. Therefore, early detection and prevention constitutes one of the four technical elements of the Global Strategy for Malaria Control [8].

Early malaria warning systems are typically based on patient records and monthly case reports provided by health authorities, who are responsible for diagnosing cases and providing effective treatments to the population. If the monitoring system is developed effectively, prevention and control measures can be performed early, allowing resources to be prioritized in high-risk areas [3,9].

Among the most used tools for the development of early warning systems are statistical prediction models based on historical case reports and indicators of environmental risks [10,11]. Quantifying the impact of environmental and climatic variables on the incidence of malaria is a crucial step toward effective early detection. However, a major limitation is the lack of access to environmental and meteorological data. For example, long-term data may be scarce, ground-based monitoring networks are often limited, and real-time data for rapid outbreak response are not always available [12,13]. In such cases, remote sensing constitutes a key source of information for the development of epidemiological predictive models in regions where data collection is limited or challenging.

In Argentina, studies using satellite information for predicting disease cases transmitted by mosquito vectors are limited and primarily focused on addressing the issue of dengue [10,14,15,16,17]. However, to date, no studies have been conducted that allow for the prediction of malaria epidemic outbreaks.

The area encompassing the Argentinean–Bolivian border is a region of intense epidemiological dynamics, where pathogenic interactions are related to structural, climatic, and social conditions, particularly given the movement of human populations in both directions. For many years, malaria transmission has been facilitated by cases imported from Bolivia [18]. Although the ArBol (Argentina–Bolivia) campaigns in the 1990s led to a reduction in malaria cases along both frontiers, adverse weather conditions hindered access to affected areas, causing a disruption in control efforts [19]. Nevertheless, the number of indigenous malaria cases decreased substantially and reached zero, and in 2019, Argentina was officially recognized by the World Health Organization (WHO) as free from autochthonous malaria transmission.

In this context, this study analyzes the influence of environmental factors (obtained from remote sensing or satellite imagery) on the incidence of malaria, with the goal of developing models for future epidemic outbreaks and maintaining epidemiological surveillance in San Ramón de la Nueva Orán, Orán Department (Salta Province). To achieve this, epidemiological data from malaria cases in San Ramón de la Nueva Orán between 1986 and 2005 were analyzed. A local environmental characterization of the study area was performed; environmental variables were obtained through the pre-processing and processing of satellite imagery. Remote sensing indices such as the normalized difference water index (NDWI), normalized difference vegetation index (NDVI), and land surface temperature (LST) are commonly used to distinguish different land cover types or elements based on their reflective properties across spectral bands. These indices help to enhance the detection of soil and vegetation in the visible and near-infrared spectra, while reducing the effects of terrain (slope and orientation). This information provides valuable historical–ecological context, allowing for the establishment of the relationship between environmental data and disease transmission. Furthermore, the monitoring of changes in these environmental factors, both in real time and retrospectively, is crucial for the development of early warning systems. Additionally, this study utilized ARIMA (autoregressive integrated moving average) models, integrating malaria case fluctuations and environmental variables to predict the emergence of new cases and estimate transmission risk.

## 2. Materials and Methods

### 2.1. Study Area

**Figure 1:**
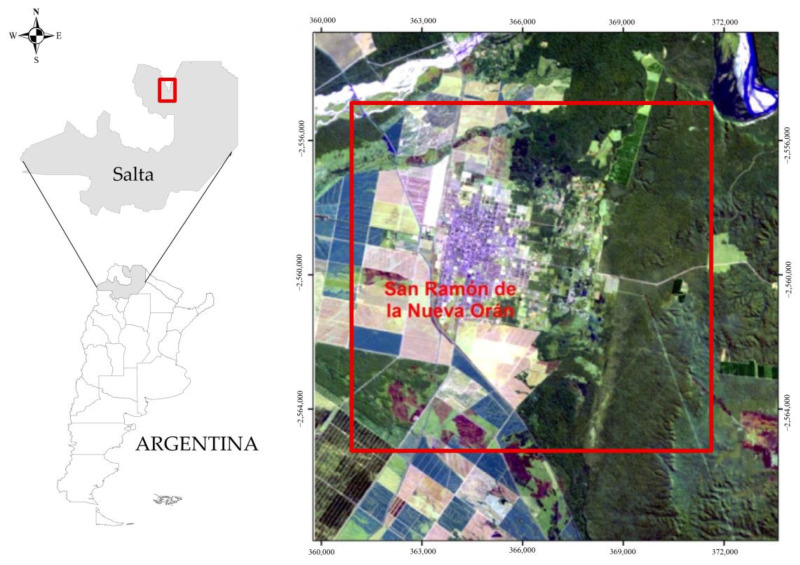
Location of the study area in the Orán Department, Salta Province, northwest Argentina.

The study area corresponds to the city of San Ramón de la Nueva Orán, located in the north of the Salta Province (Figure 1). The population is 82,413 inhabitants [20], and the city is the second most important in the province, constituting an important economic center [21,22].

The climate is subtropical, with a distinct dry season. The mean annual rainfall ranges from 700 to 1000 mm, with precipitation concentrated in the summer months (November–March) [23]. The mean annual temperature is 21.4 °C, with extreme highs reaching 45 °C during the summer. The winters are temperate, and the humidity averages around 78% [24]. The city is located in the altitudinal zone of the Yungas Piedmont forests (between 400 and 700 m above sea level (m.a.s.l.)). Land use in the region is based mainly on the cultivation of sugarcane, citrus fruits, horticulture, tropical fruit production, and logging [24].

### 2.2. Study Type

This study was descriptive and retrospective. Epidemiological sheets from the Ministry of Health of Argentina were analyzed for the 1986–2005 period. The retrospective nature of this study implies that no variables were manipulated, and no real-time tracking of individuals was conducted. Data collection was approved by the Ethical Committee of the Ministry of Health of Argentina, following the methodology described in the Protocol of the Manual de Normas y Procedimientos de Vigilancia y Control de Enfermedades de Notificación Obligatoria, Ministerio de Salud de Argentina.

### 2.3. Epidemiological Data

Technicians of the Ministry of Health of Argentina performed active campaigns to identify positive malaria cases. They visited dwellings where the people had symptoms of malaria, as well as dwellings close to positive dwellings. Before collecting samples, the technicians informed each person about the symptoms and the importance of providing a blood sample for diagnosis, using a thick blood smear (TBS) and thin blood smear to assess for the presence of the parasite. After obtaining verbal consent, the technicians collected the sample and completed the epidemiological record, which included information related to the date (day, month, year), name of the patient, address of the patient, age, sex, occupation, place of infection, previous history of malaria, the most recent movements of the individual prior to disease detection, and the classification of the case (autochthonous, introduced or imported, according to the place of infection). Data in the original sheets were digitized in Excel sheets. Malaria cases were registered monthly during the 1986–2005 period. In this study, only malaria cases reported as indigenous and introduced were used. Imported cases were excluded, under the assumption that climatic variable fluctuations at the local level potentially influenced the emergence of the former cases but not the emergence of imported cases. The fluctuation in monthly malaria cases was analyzed, with the aim of determining any seasonal disease patterns.

The variables used in this study were obtained from these epidemiological sheets with prior approval from the Ministry of Health of Argentina.

### 2.4. Landsat Images: Selection, Calibration, and Georeferencing

**Figure 2:**
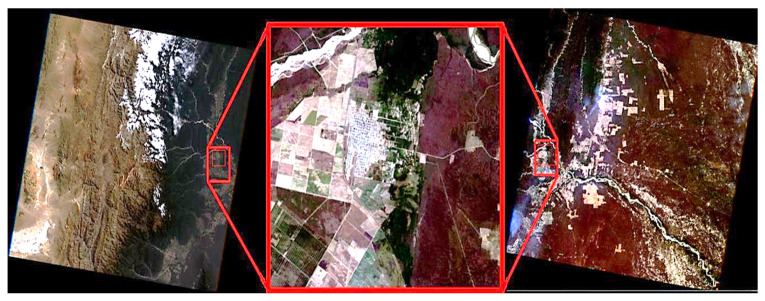
Study area (red rectangle) in two scenes of two adjacent satellite passes. Path 231 (**left**) and Path 230 (**right**), Salta Province, northwest Argentina.

Landsat data, which capture multispectral information in the visible, near-infrared, shortwave infrared, and thermal infrared wavelengths, are acquired at a 16-day overpass frequency. As the epidemiological data used in this study have a monthly resolution, a time series of Landsat satellite images with an equivalent frequency was generated (240 images from January 1986 to December 2005). Thus, an interpolation algorithm in Interactive Data Language (IDL) was implemented to estimate missing data and generate a complete time series that matched the epidemiological series. Landsat images were obtained from three different sources: the United States Geological Survey (USGS); the Global Land Cover Facility (GLCF), Maryland University, United States, with support from NASA; and the Instituto de Pesquisas Espaciais (INPE, Brazil). These sources were used to assess the accuracy of georeferencing, allowing for searches of scenes based on capture data, study area, satellite, and sensor type, among others. The study area lies at the boundary of two adjacent satellite passes (path: 230/231; row: 076) in the WRS-2 (World Reference System-2, specifically for Landsat data). Thus, it was included in both scenes, thus increasing the number of available images (Figure 2). The search was conducted at three institutions, and each image was visually analyzed to determine whether the area of interest was covered by clouds. When multiple images were available for a given month, the selection was based on temporal equidistance from the acquisition dates of the preceding and subsequent months. A total of 160 Landsat 4 and 5 TM (Thematic Mapper) images and 16 Landsat 7 ETM+(Enhanced Thematic Mapper) images were downloaded. The reference system of the images was UTM (Universal Transverse Mercator) zone 21S WGS84 (World Geodetic System).

Satellite images stored radiance values in an 8-bit format (Digital Number (DN) between 1 and 255). Therefore, calibration was required for each band to first obtain the radiance values, followed by reflectance values, for bands 1-5 and 7, along with temperature values (in Kelvin) for the thermal band 6. Three calibration methods were compared using an R script, the “Remote Sensing” R package, and “Landsat calibration” in the ENVI 4.8 program. Bands 3 and 4 of the Landsat images were calibrated using each method, and the pixel reflectance values were compared.

A script created by Dr. Anabella Ferral in 2012 was used as the calibration script in R [25]. This code was designed to calculate the spectral radiance, reflectance, and brightness temperature of LANDSAT5-TM images provided by the Comisión Nacional de Actividades Espaciales (CONAE). For bands TM1, TM2, TM3, TM4, TM5, and TM7, the output file consists of reflected images. However, for the TM6 band, it is a brightness temperature image. This script was modified and adapted for the calibration of LANDSAT 5 TM and LANDSAT 7 ETM+ images. Calibration was performed with the remote sensing package that processes Landsat, Moderate-Resolution Imaging Spectroradiometer (MODIS), Advanced Spaceborne Thermal Emission and Reflection Radiometer (ASTER), and National Oceanic and Atmospheric Administration (NOAA) (Advanced Very-High-Resolution Radiometer (AVHRR) sensor) satellite images. It includes various image pre-processing modules that calibrate, remove clouds, and calculate vegetation and water indices. The “dn2ref” and “dn2temp” functions are used to calibrate reflectance and temperature, respectively, for the Multispectral Scanner System (MSS), TM, and ETM+ sensors.

As previously mentioned, to verify the georeferencing accuracy, three satellite images from different sources were compared with Google Earth coordinates, which served as the real coordinates. By overlapping USGS Landsat images with Google Earth images, it was possible to assess the geographic correspondence between them. These Landsat images were then used as a basis for georeferencing. As the INPE images were not correctly georeferenced, georeferencing was performed for all of these images using the USGS template image. A USGS Landsat image was then used for georeferencing in ENVI 4.8 software. For this, 20 ground control points (GCPs) were selected, with a root-mean-square (RMS) error of 0.5 pixels. A first-degree polynomial was used for image adjustment, using the nearest-neighbor resampling method.

### 2.5. Normalized Indices and Environmental Variables Obtained Using Satellite Imagery

Data collected from the satellite images were processed to estimate real ecological variables and meteorological variables measured in situ [26]. These estimations involve transformations of the images using algorithms based on the bands, and they have been widely used to monitoring changes in natural factors, such as rainfall and surface temperature.

Normalized difference vegetation index (NDVI): For the identification of plant masses, the ratios or indices were based on the specific radiometric behavior of vegetation. The normalized difference vegetation index (NDVI) [27] transforms multispectral data into a single band. NDVI values vary between −1 and +1, with values closer to 1 in a given pixel or area reflecting healthier vegetation [28].

Normalized difference water index (NDWI): Different versions of the normalized difference water index exist, and all of them are considered to be good indicators of the water content and humidity conditions of vegetation. NDWI values range between −1 and 1, with water bodies exhibiting positive values and vegetation and soil exhibiting zero or negative values [29].

Three time series of Landsat images (one for the LST, one for the NDVI, and one for the NDWI) were obtained. Using the “Layer Stacking” tool of ENVI 4.8, all of the images were joined into a single file. Then, a region of interest (ROI) was defined, from which four statistics were calculated (minimum, mean, maximum value, and variance) for each time series. The ROI was delimited in a Yungas area (forest cover) where there was no change in coverage during the study period. The defined area of interest was 57 × 57 pixels (1.7 km on a side) and contained 3,249 pixels. The ROI was transformed into a .shp vector file to clip the image file. From the resulting file, four statistics of each of the three environmental variables were obtained using an IDL script created by Lic. Mario Lamfri (Com. Pers.). This script iterates through the bands of the file using i iterations and calculates the four statistics.

The missing month data were calculated using another IDL script, in which an interpolation function called /INTERPOL was used. This script calculated the missing data from a linear function using the available data.

### 2.6. ARIMA (Autoregressive Integrated Moving Average) Models, Analysis, and Adjustment

To establish the relationship between malaria prevalence and environmental variables derived from satellite imaginary, a multivariate Box–Jenkins or ARIMA model was estimated. This model was used to conduct temporal analysis of reported cases, allowing for predictions of the number of expected cases, along with confidence intervals associated with these predictions. The comparison of such predictions with the observed cases facilitates decision-making and can be used to determine whether a high number of cases corresponds to an outbreak of the disease or to random variation [30,31].

The ARIMA model included the following two parameters: autoregressive (AR), and moving average (MA), each of which included one term indicating the number of lags, designed as “degree”. Thus, the autoregressive term relates the observation obtained at the time t to the observation obtained at the times t-1 (first degree), t-2 (second degree), and so on. The moving average term relates the error (difference between observed and expected values) of time t to those of time t-1, t-2, etc. Both sets also include seasonal terms (of degree 12, 13, 52, etc., depending on the interval between observations) and their multiples [31,32].

The search for the ARIMA model that best fitted the data was performed using the “Expert modeler” option of the Temporal Series module in SPSS 15.0 software. Specifically, the series of malaria cases and environmental variables were divided into two sub-periods: one was the sub-period of estimation used to determine which model best adjusted the data, and the other was the sub-period of prediction used to test the forecast capacity of the model. The interval from January 1986 to December 1999 was selected as the optimal forecasting period because it marked the end of a period with substantial malaria cases, prior to the near-elimination of the disease. The Expert Modeler automatically searches for the model that best adjusts to each dependent series. If independent (predictor) variables are specified, the Expert Modeler selects those variables with significant associations with the dependent series for their inclusion in the ARIMA models. If the independent variables do not provide any information, a univariate ARIMA model is generated [33]. To determine the degree of adjustment of the multivariate ARIMA model proposed by the Expert Modeler, the coefficient of determination (R2) was used, which determines the proportion of the variance in the dependent variable explained by the model. This statistic value varies between 0 and 1. The adjustment of the model was also tested using residual analysis, and the lack of correlation was verified using the Ljung–Box statistical test, which assesses the existence of autocorrelation among residuals through the Q index (i.e., a non-significant Q index implies the absence of autocorrelation).

### 2.7. Influence of Environmental Variables on Malaria Cases

The degree of influence of the climatic and environmental variables obtained from the remote sensors (NDVI, NDWI, and LST) on the emergence of malaria cases in San Ramón de la Nueva Orán between January 1986 and December 2005 was analyzed.

First, a correlation analysis between the remote sensing-derived environmental variables was performed to avoid multicollinearity issues (i.e., high correlation among explanatory variables; see Table 1). Then, variable selection was performed. Two possible models were considered: Model A, which included the variables NDVImed, NDWIvar, LSTmed, and LSTvar; and Model B, which included the variables NDWImed, NDVIvar, LSTmed, and LSTvar.

Before applying the statistical analyses to Models A and B, an exploratory analysis of the data was performed to assess for correlations between the number of malaria cases and the independent variables. The analysis revealed that the number of infected patients was higher when the mean independent variables with a one-month lag were considered.

On the other hand, analysis of the annual mean and variance of the independent variables revealed that the mean number of malaria cases exhibited higher annual variability, and that the mean and variance of the total number of cases differed throughout the 20-year period. This result led to the application of hyper-Poisson regression models for overdispersed data, with a variable ⎣ parameter (incidence rate) by year (i.e., ⎣ is not constant but varies over time). In summary, the fluctuations in the climatic and environmental variables in relation to the emergence of malaria cases were analyzed through Poisson regressions.

The incidence rate ratio (IRR) was used to determine the percentage of influence of each of the environmental/climatic variables on the emergence of malaria cases. In addition, standard error (SE), *p*, and confidence interval values (95% CIs) were obtained.

In the present study, data were analyzed using the aforementioned Poisson regressions, along with the two previously described Models A and B (including NDVImed, NDWIvar, LSTmed, and LSTvar and NDWImed, NDVIvar, LSTmed, and LSTvar, respectively).

## 3. Results

### 3.1. Fluctuation of Cases in San Ramón de la Nueva Orán

**Figure 3:**
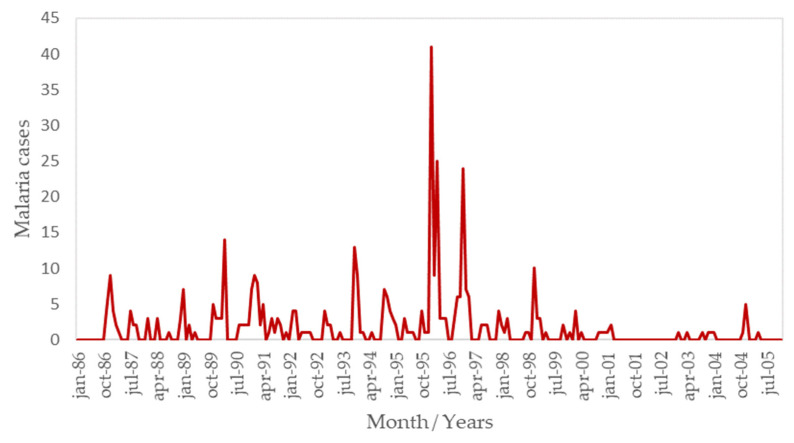
Temporal fluctuations in autochthonous/introduced malaria cases reported in San Ramón de la Nueva Orán, Salta Province.

The fluctuation in indigenous/introduced malaria cases from January 1986 to December 2005 was analyzed to determine the existence of a seasonal disease pattern. The total number of reported indigenous/introduced malaria cases during the 20-year period in San Ramón de la Nueva Orán was 453. The highest peaks occurred in 1996, during January, February, and April, with 41, 24, and 25 cases, respectively. Another important peak was observed in February 1997, with 24 cases. There were years with no transmission of malaria, such as 1986. In 2000, the number of reported cases was near zero, with sporadic occurrences of only one or two cases in the summer months. A total of 23 cases were identified from January 2000 to December 2005 (Figure 3). A seasonal pattern was observed when analyzing the occurrence of malaria cases in relation to the months of the year, with most cases reported during the summer months (December–March).

### 3.2. Descriptive Analysis of Malaria Cases During the 1986–2005 Period

The epidemiological characteristics and relative frequencies of malaria cases were determined. Descriptive graphics for each of the variables considered (such as “sex”, “age group”, and “occupation”) were generated using Excel software v16.0.

The annual frequency of the occurrence of malaria cases based on sex was analyzed during the period of 1986–2005. It was observed that, in all years, most cases (more than 50%) corresponded to male individuals. Considering the age of the infected patients during the same period, a higher frequency of infected individuals was observed in the age group between 15 and 30 years old, with 143 reported cases, followed by 127 cases in the age group between 31 and 45 years old, and 96 cases in the age group between 0 and 15 years old.

Lastly, the frequency of people infected with malaria was assessed based on their different types of work. Infected individuals were grouped according to whether their work activity was performed in rural areas (countryside) or urban areas (city). Underage individuals and schoolchildren, who did not work, were grouped as minors. In total, 183 malaria cases were noted in the countryside group, and 169 cases in the city group. A much lower proportion was noted in the minor group.

### 3.3. Results of ARIMA Models

The model proposed by the Expert Modeler for the prediction of malaria cases, based on the environmental variables NDVI, NDWI, and LST extracted from satellite images, corresponded to an ARIMA model of (0,0,1) (0,0,0). This indicates that the variables did not require any differentiation, as the model would have automatically included such steps if necessary. This type of ARIMA model is equivalent to a moving-average model AR (1), where the cases in a given month depend on the error from the previous month.

The Expert Modeler was set to search for the best ARIMA model, taking into account the 12 satellite-derived environmental variables as predictor variables. It identified two significant predictor variables (“number of predictors”). The coefficient of determination was 0.882, indicating that 88% of the observed variability could be explained by the model. The validity of the model was confirmed through the visualization of the residuals, with no autocorrelation for any lag, as corroborated by the Ljung–Box test, which was not significant (*p* = 0.420) (Table 2).

It was observed that the maximum NDVI and mean NDWI were the only environmental variables that influenced the emergence of malaria cases in San Ramón de la Nueva Orán. It was also observed that the effects of the mean NDWI did not seem to be immediate but, rather, occurred after 5 months (Table 3).

**Figure 4:**
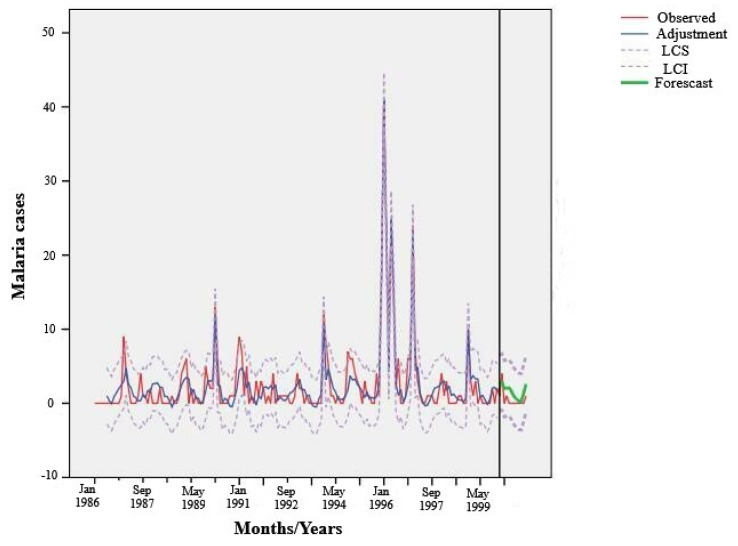
Adjustment of the ARIMA model (0,0,1), (0,0,0) and prediction of malaria cases in San Ramón de la Nueva Orán from 1986 to 2000.

Figure 4 shows the fluctuation of malaria cases during the period between January 1986 and December 2000 (red line). The adjustment of the proposed model (blue line), the upper and lower confidence intervals (dotted lines), and the fluctuation in cases proposed by the model for the year 2000 (in green) can also be observed. Figure 4 shows that the ARIMA model (0,0,1) presented a good adjustment with regard to malaria cases during the period of estimation, and it was also capable of predicting malaria cases that occurred during the prediction period (year 2000).

### 3.4. Influence of Environmental Variables on Malaria Cases

#### 3.4.1. Model A

According to the variables included in Model A (NDVImed, NDWIvar, LSTmed, and LSTvar), it was observed that only NDVImed and LSTmed were significant (*p* = 0.001 and *p* = 0.016, respectively) (Table 4).

**Figure 5:**
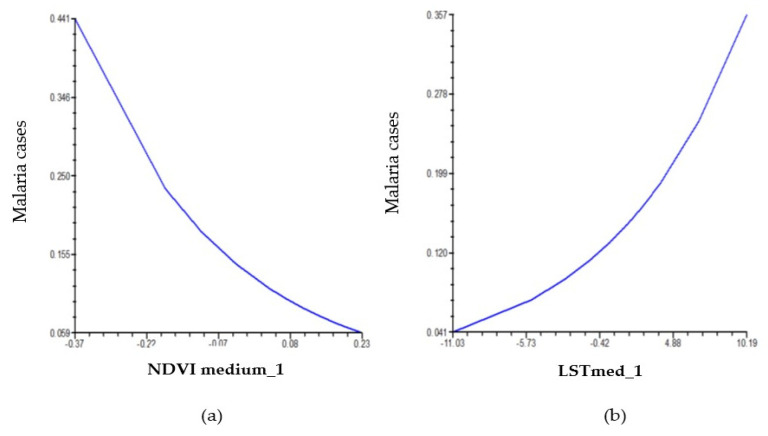
Model A: Relation between malaria cases and observed variability in (**a**) mean NDVI and (**b**) mean LST.

Using Model A, the following was predicted:

(1) For each 0.1 unit increase in NDVImed with respect to the NDVImed of the 20-year period (0.57318), there is a 29% decrease in IRR (in 0.71) compared to the incidence rate that occurs when NDVImed and LSTmed reach the 20-year mean values (0.57318 and 19.9089, respectively).

(2) For each unit of change in LSTmed with respect to the mean LSTmed of the 20-year period (19.9089), the IRR changes by 1.107 (11% increase) compared to the incidence rate that occurs when NDVImed and LSTmed reach the 20-year mean values (0.57318 and 19.9089, respectively) (Figure 5).

#### 3.4.2. Model B

Of the variables included in Model B (NDWImed, NDVIvar, LSTmed, and LSTvar), only NDWImed and LSTmed were significant (*p* = 0.003 and *p* = 0.005, respectively) (Table 5).

**Figure 6:**
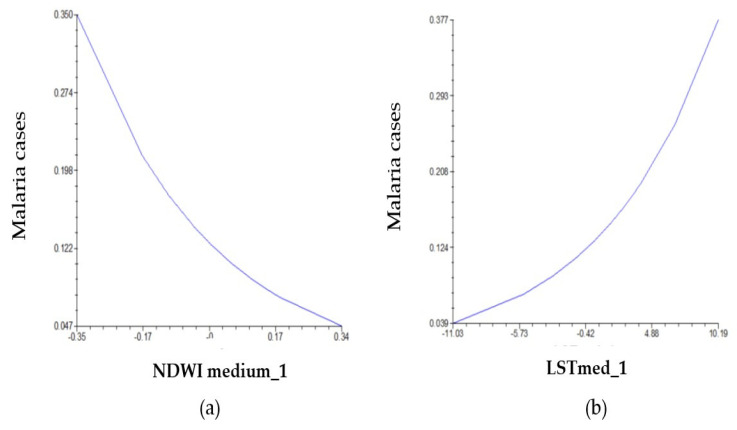
Model B: Relation between malaria cases and observed variability in (**a**) mean NDWI and (**b**) mean LST.

Using Model B, the following was predicted:

(1) For every 0.1 unit increase in NDWImed with respect to the 20-year mean NDWImed (0.25908), IRR is reduced by 25% compared to the incidence rate that occurs when NDWImed and LSTmed reach the 20-year mean values (0.25908 and 19.9089, respectively).

(2) For every unit of change in LSTmed with respect to the 20-year mean LSTmed (19.9089), the IRR increases by 11% compared to the incidence rate that occurs when NDWImed and LSTmed reach the 20-year mean values (0.25908 and 19.9089, respectively) (Figure 6).

Both models (A and B) showed very similar results, given that the effects of vegetation and humidity were similar; however, Model A presented a higher reliability.

## 4. Discussion

Epidemiologically, northwestern Argentina is an important region, given the malaria outbreaks recorded since the late 19th century. The development of the disease was influenced by topography, climate, and phytogeography [18]. As a result, the “malaric mountainous area” was limited to the northern and central provinces of the country [34,35]. Historically, the disease transmission season occurred in October or November in Salta and Jujuy; in November or December in Tucumán, Santiago del Estero, and Catamarca; and in December and January in La Rioja and Córdoba. However, it was delayed if the previous winter and spring were especially cold and rainy, respectively. The transmission of the disease could last until May or June throughout the entire area, and it could be extended during temperate winters [18,34,36].

In the current study, the fluctuation in malaria cases in San Ramón de la Nueva Orán during the 1986–2005 period showed a seasonal pattern, with peaks of abundance during the summer and autumn months. This finding is consistent with those of authors who found that malaria cases emerged during summer and autumn in Aguas Blancas (on the border with Bolivia), preceded by the highest abundance of the mosquito vector *An. pseudopunctipennis* 3 months prior [18]. Thus, the emergence of malaria cases was related not only to the presence of the mosquito but also to the periods of its highest abundance, which occurred in spring [37,38] and summer [37] in the study area.

San Ramón de la Nueva Orán is located in the Yungas Piedmont, an area that has suffered severe anthropic transformations in recent decades, mainly due to deforestation from agricultural expansion or urbanization. The latter is linked to the migration of the population toward rural areas in search of work. This increases contact with the mosquito populations and the risk of contracting the disease by rural workers, who generally do not use body-covering clothing or repellents during their working hours. The contact occurs because the “edge effect” of natural vegetation patches increases in areas adjacent to agriculture, which are characterized by a high abundance of mosquitoes [39]. It is important to consider that the emergence of agricultural land generates suitable conditions for the generation of breeding grounds, such as irrigation channels.

Based on the descriptive analysis, the most affected group comprised males between 15 and 30 years old, followed by the 31- to 45-year-old group. This is logical, because young male adults generally perform rural work, while women and children remain in the household. This is consistent with the findings of a study performed in Perú, where the authors surveyed people who had contracted the disease, revealing that all were male farm workers who were not protected during working hours [40]. Dantur Juri et al. [18] also found a high prevalence (79%) of male cases in El Oculto compared with Aguas Blancas (49%). The authors attributed the difference to the fact that Aguas Blancas is on the border with Bolivia; thus, trade is the main commercial activity, and women are also involved. It is well known that the dynamics of malaria are related to human activities [41,42,43].

As previously mentioned, this should be taken into account by medical authorities when implementing control measurements. Through identifying seasons with a higher number of reported cases, it can be inferred that exposure and infection risk are also elevated during these periods. In addition, it is crucial to inform the target population so that they can implement adequate control measures to avoid contracting the disease by avoiding contact with the vector.

The indices derived from the satellite images and ARIMA time-series models were developed to predict the emergence of new malaria cases and to estimate the risk of disease transmission. In the present study, the ARIMA model was adjusted to the changes in the epidemiological behavior of malaria in San Ramón de la Nueva Orán, and these changes were generated automatically. Using the NDVI, NDWI, and LST indices as predictor variables, it was observed that the case prevalence was related to the emergence of cases in the previous month, along with the maximum NDVI and the mean NDWI observed five months prior.

The relationship between the maximum NDVI and malaria prevalence has been previously reported. In that study, the vegetation cover directly affected the abundance of anopheline mosquitoes [44], because it maintained soil and air humidity and provided more shelter for the mosquitoes [18]. Another study performed in Kenya reported similar results. The study observed that the monthly number of malaria cases was strongly correlated with the NDVI of the previous month. In turn, the authors observed that, in the context of a minimum NDVI value, the number of malaria cases recorded in the following month was greater than 5% of the total [26]. In Burundi (Africa), a model was developed to predict the incidence of malaria from the association between climatic variables and NDVI, with the cases of malaria reported monthly. The best model to predict the incidence of the disease included the rainfall, maximum temperature, and NDVI of the previous month. In addition, the relationships between monthly records of malaria cases from 23 provinces of Afghanistan and environmental variables, such as rainfall, temperature, and NDVI, were reported in another study [45]. Adimi et al. [46] found that the NDVI was the best predictor of malaria’s incidence. Similar results were reported by Gaudart et al. [47] in a study conducted in Mali, Africa, in which the authors established a cohort of children up to 12 years old and followed them for 5 years to evaluate parasitemia in their blood samples. Through a time-series analysis associating the incidence of *Plasmodium falciparum* and the NDVI, the authors found that this index explained the seasonal pattern of the parasite with a lag of 15 days, and that an increase in the NDVI generated a significant increase in parasitemia.

As previously cited, in this study, it was observed that the NDWI was inversely related to the emergence of malaria cases in San Ramón de la Nueva Orán. The use of the NDWI as a regressor variable in the temporal and spatial models of different diseases has been previously reported by some authors. Estallo et al. [16] demonstrated that the NDWI was a good predictor of the household infection index (HI) in San Ramón de la Nueva Orán. In addition, Cohen et al. [48] used the NDWI, among other remote sensing-derived environmental variables, to develop spatial models of malaria transmission in Swaziland, Africa. The authors showed that the transmission of the disease was related to areas with high NDWI values. Dambach et al. [49] showed a significant relationship between the NDWI and the presence of breeding sites of malaria vectors in a rural area of Burkina Faso. This finding was of great importance for the generation of risk maps for disease transmission. In Italy, Rosa et al. [50] analyzed a time series of captures of *Culex pipiens*, a mosquito vector of the West Nile virus, and environmental variables such as rainfall, temperature, and NDWI. They found that an increase in NDWI at the beginning of the year was related to a shortening of the season of the highest abundance of mosquitoes. These results are consistent with those reported by Estallo et al. [16] and Machault et al. [51]. The NDWI is used for environmental characterization because it measures the foliar content of water in vegetation. It also constitutes an indirect measure of rainfall and soil humidity, which play important roles in the biology of disease vectors.

In the described studies, the relation between the NDWI and the drivers of the emergence of several diseases was positive. However, in this study, the NDWI exhibited a negative effect on the emergence of malaria cases 5 months later. Given that the NDWI is related to the occurrence of rainfall and humidity conditions, the inverse relationship found here between the NDWI and the emergence of malaria cases might be explained by the occurrence of heavy rains, which could wash away the breeding sites of the immature forms of the mosquito vectors. This would subsequently reduce the population of anopheline mosquitoes and indirectly reduce the number of malaria cases.

A similar result was reported in a study conducted in Colombia, where the transmission of dengue was associated with variability in local climatic conditions, with a lag of 20 weeks (approximately 5 months) in the case of rainfall [30]. According to the authors, one of the hypotheses proposed to explain this lag was that local populations of the vector might connect through the dispersion of a small group of migrant females that could colonize recently established new habitats (water reservoirs) during periods of higher rainfall. Subsequently, due to the generation of many populations of the vector, the flux of individuals within the new habitats is possibly favored, ultimately facilitating the long-term persistence of vector populations. This can generate a critical population density of the vector, which likely allows for the efficient dispersion of dengue virus within human populations.

Using multilevel Poisson regressions and the two formulated models, it was observed that malaria cases potentially increase with both a decrease in mean NDVI and an increase in mean LST, or with both a decrease in NDWI and an increase in mean LST. In other words, in both formulated models, malaria cases were positively related to an increase in mean LST. It is known that temperature is a key factor for malaria transmission [52].

Several authors [42,44,53,54] have reported that an increase in temperature led to faster hatching of eggs and shortened the duration of the larval period, generating a higher number of adults in a shorter period of time, thus increasing the populations of anophelines. Furthermore, with the increase in temperature, the gonotrophic cycle of the female mosquito shortens, which increases the frequency of blood intakes, i.e., the biting rate [41,42,54]. Lindblade et al. [41] not only demonstrated that an increase in temperature resulted in an increase in the number of adults of *Anopheles (Cellia) gambiae* found in households, but also found that the biting rate was higher in localities with higher temperatures. On the other hand, different laboratory tests demonstrated a reduction in the gonotrophic cycle of *An. albimanus* when exposed to temperatures between 24 and 30 °C [55].

In the study by Afrane et al. [56], the authors reported a reduction in the gonotrophic cycle of *An. gambiae* from 0.9 to 1.7 days in localities of Iguhu (Uganda) with higher temperatures. In addition, the extrinsic incubation period of the parasite was directly related to temperature. Specifically, at increasing temperatures, the incubation period of the *Plasmodium* parasite is shortened [42,44,53,54,56], subsequently increasing the effective lifetime of the vector. Thus, for example, the extrinsic period of *Plasmodium falciparum* in *An. gambiae* was reduced by 17.3 days (from 55.5 to 38.2 days) when the temperature increased from 18 to 18.9 °C [41]. In general terms, in the case of the mosquito, the gonadotrophic cycle lasts 2–3 days, matching with the biting of females in search of blood [53].

The relationship between the emergence of malaria cases and temperature has been previously reported in the country by Dantur Juri et al. [18], who showed that malaria cases in the El Oculto and Aguas Blancas localities (Orán Department, Salta Province) were associated with the mean and maximum mean temperatures, among other variables. In El Oculto, increases in the maximum mean temperature provoked an increase in the emergence risk of malaria cases. In Aguas Blancas, the risk of malaria increased with increased mean monthly temperature and relative humidity. In addition, Sáez-Sáez et al. [54] analyzed the relationship between rainfall and temperature with respect to the incidence of the disease in Sucre (Venezuela). In the aforementioned study, malaria cases and climatic variables showed a positive correlation, and rainfall and air temperature were identified as the variables that best explained the emergence of malaria.

In Model A of the present study, the cases of malaria were also influenced by the decrease in the mean NDVI. In Model B, malaria cases were also affected by a decrease in the mean NDWI. This might be due to the fact that both indices are related. The NDVI measures the status of the vegetation, including its vigor and “greenness” in relation to the process of photosynthesis. This, in turn, is positively related to environmental conditions such as rainfall, humidity, and temperature. The NDWI measures the water content of vegetation and is thus an indirect indicator of soil and environmental humidity. Ultimately, an increase in rainfall will produce increases in both the NDWI and NDVI. It is well known that rainfall generates suitable conditions for the creation of new larval habitats; however, strong rainfall might produce floods that wash away the breeding sites of anophelines [18].

Studies have reported results that differ from those obtained in the present work, namely, a positive relationship between the NDVI and malaria cases. However, in a study performed in Bangladesh, Haque et al. [57] analyzed malaria cases in relation to meteorological and environmental variables, such as rainfall, temperature, humidity, and the NDVI. The authors reported that an increase of 0.1 in monthly NDVI was associated with a 30.4% decrease in malaria cases.

It is important to recognize that the dynamics of malaria are highly complex, influenced not only by environmental factors but also by social, economic, and political conditions. Household quality (which is closely linked to the population’s economic status), hygiene conditions, actions performed by public health agencies, population dynamics, and migration within areas of endemic transmission are factors that directly and indirectly affect the spread of the disease.

The relevance of this retrospective framework with historical data provides valuable information, despite the country’s certification as free of autochthonous malaria transmission in 2019. This study not only constitutes the only precedent on this topic to date but also lays the groundwork for future research in the country. Its perspective is crucial for determining patterns, trends, and specific risk factors, both environmental and social, from data related to the historical dynamics of the disease. The comparison of malaria dynamics before certification reveals changes in population susceptibility and the effectiveness of interventions, allowing for adjustments in health policy strategies. This information is fundamental for developing long-term control and surveillance plans, especially in the context of imported cases and disease reintroduction.

## 5. Conclusions

In San Ramón de la Nueva Orán, a seasonal fluctuation in malaria cases was observed during the 1986–2005 period, with a higher number of infected individuals reported during the summer months. In addition, the emergence of malaria is potentially related to rural activities performed by young male adults (15–45 years old). The multivariate ARIMA model proved to be useful for predicting malaria cases in San Ramón de la Nueva Orán, where the environmental variables related to the emergence of cases included the maximum NDVI and mean NDWI, with a lag of 5 months for the latter, according to the models. Additionally, the relationships between climatic and environmental factors and malaria cases were quantified using multilevel Poisson regression models, based on incidence rate ratio (IRR) calculations. This analysis showed that malaria cases were associated with an increase in mean temperature and a decrease in NDVI.

## Figures and Tables

**Table 1:** Correlation analysis between the environmental variables NDVI, NDWI, and LST.

	NDVImin	meanNDVI	NDVImax	NDVIvar	NDWImin	Wimedia	NDWImax	NDWIvar	LSTmin	meanLST	LSTmax
**NDVImin**	1										
**meanNDVI**	0.865	1.000									
**NDVImax**	0.759	0.958	1.000								
**NDVIvar**	−0.434	−0.250	−0.110	1.000							
**NDWImin**	0.823	0.768	0.665	−0.503	1.000						
**Mean NDWI**	0.779	0.905	0.862	−0.309	0.876	1.000					
**NDWImax**	0.582	0.807	0.828	−0.189	0.770	0.932	1.000				
**NDWIvar**	−0.419	−0.141	0.081	0.194	−0.247	−0.051	0.171	1.000			
**LSTmin**	0.128	0.072	0.032	−0.019	0.055	0.057	0.010	−0.085	1.000		
**meanLST**	−0.010	−0.026	−0.056	0.058	−0.042	−0.042	−0.049	−0.029	0.961	1.000	
**LSTmax**	−0.093	−0.095	−0.110	0.123	−0.128	−0.115	−0.110	0.006	0.932	0.989	1.000
**LSTVar**	−0.544	−0.409	−0.317	0.410	−0.453	−0.411	−0.269	0.304	−0.185	0.064	0.156

NDVI: normalized difference vegetation index; NDWI: normalized difference water index; LST: land surface temperature.

**Table 2:** Details of the parameters of the model proposed by the Expert Modeler.

Model	Number of Predictors	Statistics of Model Adjustment				Ljung–Box Q (18)		Number of Outliers
		R-Squared Stationary	R-Squared	RMSE	Statistics	Degrees of Freedom	Sig.	
**Cases-Model_1**	2	0.882	0.882	1.856	17.511	17	0.420	9

**Table 3:** Estimators of TF proposed by the Expert Modeler.

					Estimation	SE	t-Value	*p*-Value
**Cases-Model_1**	**Cases**	**Without transformation**	**MA**	Delay 1	0.280	0.081	−3.473	0.001
	**NDVI_max**	**Without transformation**	**Numerator**	Delay 0	4.368	0.453	9.639	0.000
	**NDWI_mean**	**Without transformation**	**Delay**		5			
			**Numerator**	Delay 0	−4.588	0.963	−4.764	0.000

SE: standard error.

**Table 4:** Model A estimators.

Variable	IRR	SE	*p*	95% CI
**Mean NDVI**	0.035320	0.927147	0.001	(0.006–0.219)
**Mean LST**	1.107243	0.041824	0.016	(1.020–1.202)

NDVI: normalized difference vegetation index; LST: land surface temperature; IRR: incidence rate ratio; SE: standard error; 95% CI: 95% confidence interval.

**Table 5:** Model B estimators.

Variables	IRR	SE	*p*	95% CI
**Mean NDWI**	0.053598	0.999834	0.005	(0.006–0.318)
**Mean LST**	1.112393	0.037246	0.013	(1.034–1.198)

NDWI: normalized difference water index; LST: land surface temperature; IRR: incidence rate ratio; SE: standard error; 95% CI: 95% confidence interval.

## Data Availability

The original contributions presented in this study are included in the article. Further inquiries can be directed to the corresponding author.
